# Application of GC-IMS coupled with chemometric analysis for the classification and authentication of geographical indication agricultural products and food

**DOI:** 10.3389/fnut.2023.1247695

**Published:** 2023-09-01

**Authors:** Hong Zhu, Dazhou Zhu, Junmao Sun

**Affiliations:** Institute of Food and Nutrition Development, Ministry of Agriculture and Rural Affairs, Beijing, China

**Keywords:** geographical indication, GC-IMS, chemometric analysis, PLS-DA, aroma

## Abstract

Geographical indications (GI) are used to protect the brand value of agricultural products, foodstuffs, and wine and promote the sustainable development of the agricultural and food industries. Despite the necessity for the traceability and recognition of GI product characteristics, no rapid, non-destructive approaches currently exist to identify, classify, and predict these properties. The application of gas chromatography-ion mobility spectrometry (GC-IMS) has increased exponentially due to instrument robustness and simplicity. This paper provided a detailed overview of recent GC-IMS applications in China for the quality evaluation of GI products and food, including agricultural products, as well as traditional Chinese food and liquor. The general workflow of GC-IMS coupled with chemometric analysis is presented, including sample collection, model construction and interpretation, and data acquisition, processing, and fusion. Several conclusions are drawn to increase partial least squares-discriminant analysis (PLS-DA) model precision, a chemometric technique frequently combined with GC-IMS.

## 1. Introduction

Geographical indication (GI) protection is crucial for safeguarding the brand value of agricultural goods, food, and wine and furthering the sustainable growth of these industries. Although the multiple Chinese GI protection schemes have been governed by different agencies before 2022, these strategies are now managed by the China National Intellectual Property Administration. Moreover, the China-European Union (EU) GI agreement came into effect on March 1st, 2021, promoting bilateral trade in agricultural products and food between China and the EU. One hundred GIs from both the EU and China, including liquor, tea, foodstuff, and agricultural products, received protection, providing consumers with peace of mind regarding the authenticity of products. Furthermore, each side is expected to add an additional 175 products to the GI list. Although GI protection presents significant economic benefits to enterprises and governments, it is often accompanied by fraud incidents that target higher-priced products. For example, in the rice industry, Wuchang rice represents high-quality GI rice due to the excellent natural conditions in the northeast region of China. However, recent years have seen severe food fraud cases related to Wuchang rice, with adulterated rice accounting for 90% of the market.

In addition to the area of origin, many other GI product characteristics require accurate identification, which can be derived from the GI product history, including traditional processing procedures, indigenous seeds, and animal breeds. Furthermore, the market demand for high-quality agricultural products and food validates the abundant supply of GI goods that meet consumer requirements. For example, feeding regimens are crucial for GI beef and lamb. Lamb from Xilinguole and Hulunbeier (pastoral areas in Inner Mongolia), favored by Chinese consumers, is famous for its grazing and grass-feeding regimen. Moreover, since most GIs in China are labeled according to the geographical area, it is difficult for both Chinese and overseas consumers to identify them as indicators of qualities or unique characteristics. This can create confusion, and consumers are inclined to only consider GIs indicative of the place of origin, disregarding the qualities, characteristics, or reputation.

Because of the necessity for the traceability and recognition of GI product characteristics, several rapid, non-destructive approaches currently exist to identify, classify, and predict these properties, such as near-infrared spectroscopy and Raman spectroscopy. Other than spectroscopic techniques, GC-IMS may be a viable alternative to traditional flavor analysis methods like chromatography olfactometry (GC-O), electronic nose (E-nose), and gas chromatography-mass spectrometry (GC-MS). Instrument robustness and simplicity has exponentially increased the application of gas chromatography-ion mobility spectrometry (GC-IMS) for food authentication, processing, storage monitoring, illegal additive identification, and harmful compound detection (1). One benefit of GC-IMS over traditional assessment methods, like GC-MS, for recognizing volatile organic compounds (VOCs) is that it operates at atmospheric pressure without the use of vacuum pumps (2), while the portable ionization source allows on-site real-time detection. Furthermore, GC-IMS presents a substantial advantage in identifying isomeric molecules, specifically ring-isomeric compounds (3).

Combining GC-IMS GI product fingerprinting with chemometric methods is widely used for the identification of quality, adulteration, and fraud in products such as Jingyuan lamb (4), Wuchang rice (5), Fu brick tea (6), Shaoxing yellow wine (7), and Iberian dry-cured ham (8). Chemometrics models, such as partial least squares discriminant analysis (PLS-DA), are commonly used for sample classification. The outstanding advantage of this technique is the ability to recognize subtle gaps in similar samples.

As far as is known, minimal studies are available regarding the utilization of GC-IMS in GI products, while a systematic summary involving the workflow of GC-IMS combined with a chemometrics model is lacking. This paper reviews the GC-IMS application to various GI agricultural and food products in China, including rice, red meat, fruit, oil seed, honey, fish, spice, tea, dry-cured ham, Chinese yellow wine, and Baijiu to authenticate various factors, including place of origin, harvest season, animal age, and feeding regimens. The aim of this research is to conclude a standard operating procedure for GC-IMS couple with chemometric methods on GI products classification and authentication, and to dig deeper into the factors affecting the accuracy of PLS-DA models.

## 2. GC-IMS strategies for GI protection

### 2.1. The general workflow for the traceability and identification of GI product characteristics

**Figure 1:**
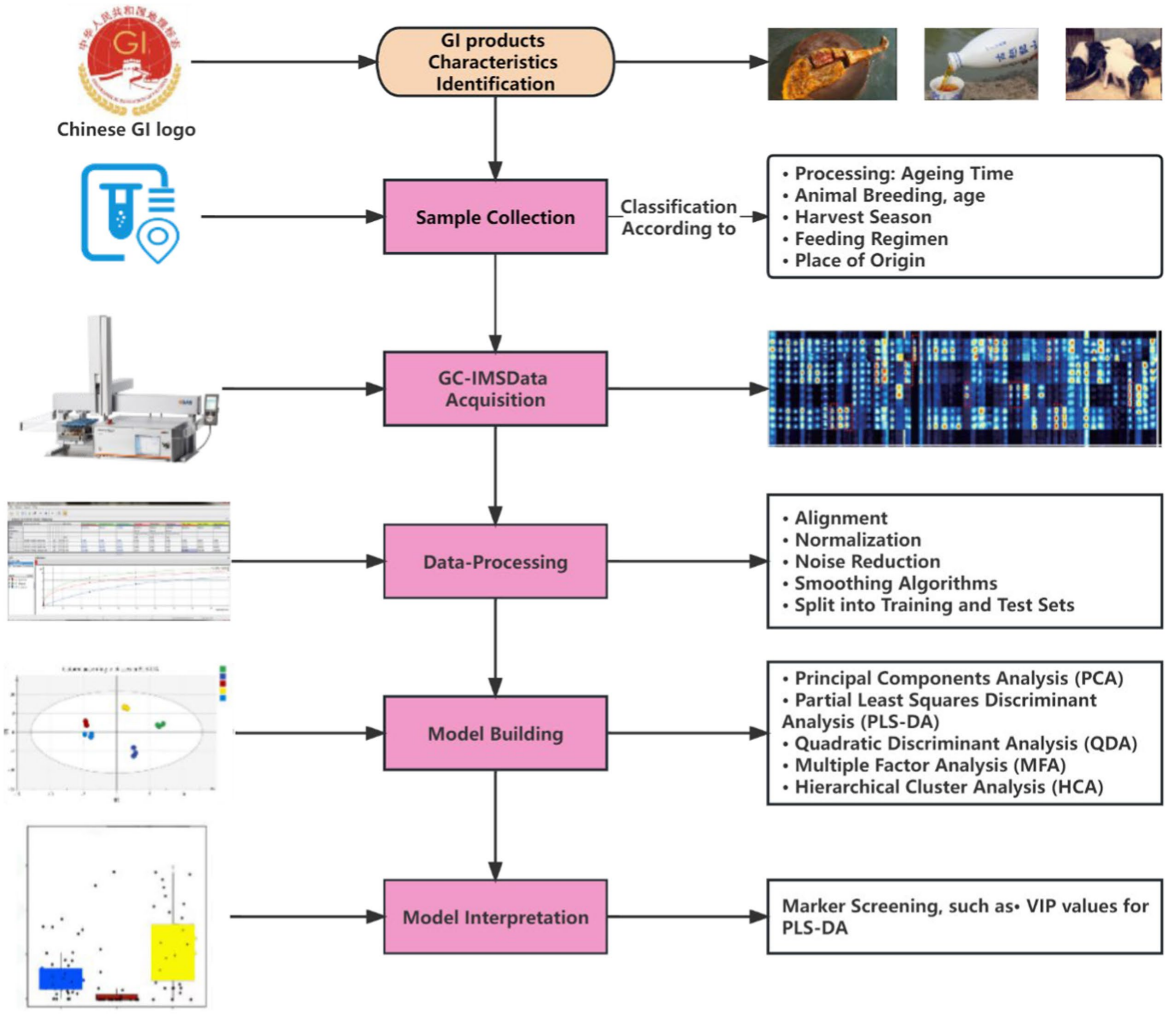
General workflow for the traceability and identification of GI product characteristics.

Figure 1 presents the general workflow for the traceability and identification of GI product characteristics using the combined GC-IMS and chemometrics method. Several classical researches are listed in Table 1. Sample collection represented the first step in this process, during which traceability, precision, and variety were more important than the number of samples used. For example, the importance of information related to sample origin and harvest season equaled that of category. Therefore, samples with limited information could not increase classification accuracy, while experimental errors or labeling mistakes led to outliers.

**Table 1:** Recent studies involving the combination of GC-IMS with chemometric methods for GI production in China.

Reference	GI characteristics	Number of samples	IMS types (ionization source)	Separation	Chemometrics method on GC-IMS data	Complementary analysis	Method and number of compounds identification	Data-split; (cross-) validation
Chen et al. (5)	Geographical origin	Rice (53)	FlavourSpec^®^ by GAS Tritium (6.5 KeV)	MXT-5 (15 m × 0.53 mm × 1 μm)	PCA; QDA	No	46, GC-IMS library	28/25
Wang et al. (4)	Animal age	Female lambs (18)	FlavourSpec^®^ by GAS	SE-54 (15 m × 0.53 mm × 1 μm)	PCA	No	66, GC-IMS library	No
Wu et al. (9)	Animal breed	Pigs (18)	FlavourSpec^®^ by GAS	RTX-5 (15 m × 0.53 mm × 0.1 μm)	PCA	GC-O-MS	59, GC-IMS library	No
Feng et al. (10)	Geographical origin	Grapes (6)	FlavourSpec^®^ by GAS	FS-SE-54-CB-1 (15 m × 0.53 mm × 1.0 μm)	PCA	Sensory analysis	36, GC-IMS library	No
Mi et al. (11)	Geographical origin	Sesame seeds (15)	FlavourSpec^®^ by GAS	MXT-5 (15 m × 0.53 mm × 1 μm)	PCA; PLS-DA	No	44, GC-IMS library	Cross-validation
Wang et al. (12)	Harvest season	Honey (120)	FlavourSpec^®^ by GAS	FS-SE-54-CB-0.5 (15 m × 0.53 mm)	PCA; PLS-DA	No	25, GC-IMS library	Cross-validation
Duan et al. (13)	Geographical origins	Salmonid (12)	FlavourSpec^®^ by GAS	FS-SE-54-CB-1 (15 m × 0.53 mm × 1.0 μm)	PCA; HCA	E-nose	35, GC-IMS library	No
Feng et al. (14)	Geographical origins	Huajiao (8)	FlavourSpec^®^ by GAS	FS-SE-54 (15 m × 0.53 mm)	PCA; PLS-DA	GC-MS, E-nose	49, GC-IMS library	Cross-validation
Xiao et al. (6)	Geographical origins	Fu brick tea (15)	FlavourSpec^®^ by GAS	MXT-5 (15 m × 0.53 mm)	PCA, PLS-DA, HCA	GC-MS	63, GC-IMS library	No
Yang et al. (15)	Aroma type	Tea (more than 80)	FlavourSpec^®^ by GAS	MXT-5 (15 m × 0.53 mm × 1 μm)	PLS-DA	E-nose	38, GC-IMS library, user-built database	Cross-validation
Liu et al. (16)	Harvest season	*Yingde* black teas (9)	FlavourSpec^®^ by GAS	FS-SE-54-CB (15 m × 0.53 mm)	No	GC-O-MS	68, GC-IMS library	No
Li et al. (17)	Geographical origins	Chinese dry-cured hams (6)	FlavourSpec^®^ by GAS	FS-SE-54-CB-1 (15 m × 0.53 mm)	PCA; MFA	GC × GC-ToF-MS	45, GC-IMS library	No
Liu et al. (18)	Aging time	*Jinhua* ham (6)	FlavourSpec^®^ by GAS	SE-54 (15 m × 0.53 mm × 1 μm)	PCA	E-nose	37, GC-IMS library	No
Chen et al. (7)	Geographical origins	Chinese yellow wine (122)	FlavourSpec^®^ by GAS	Non-polar column	PCA; QDA	No	16; GC-IMS library	79/43
Chen et al. (19)	Aging time	Baijiu (39)	FlavourSpec^®^ by GAS	DB-FFAP (60 m × 0.25 mm × 0.25 μm)	PLSR	No	93; pure standards	35/4; 7-fold cross-validation

The second step involved data acquisition, including VOC extraction and separation via GC-IMS. The GC-IMS plot was two-dimensional, with the GC retention and IMS drift times representing separate parts. GI agricultural and food product variability was demonstrated as VOC profiles or fingerprints (20). The data acquisition was evaluated according to the suitability for factorial analysis, as determined via the Kaiser-Meyer-Olkin (KMO) test, which measured the differences between the variables.

The third step involved data processing. There is a need to use a reference substance for alignment, especially when long separation columns were equipped by GC-IMS. The average of several measurements was determined, while the background noise was removed. The data sets were normalized using scaling methods like unit variance, mean centering, and Pareto scaling. Smoothing algorithms, including Gaussian and Savitzky-Golay smoothing, were employed for further noise reduction (21). Finally, the data set was divided into test and training sets for supervised analyses.

The fourth step involved creating a model utilizing regression techniques for categorization, exploratory analysis, and quantitative assessment. Exploratory, unsupervised methods, like hierarchical cluster analysis (HCA) or principal component analysis (PCA), are commonly utilized for pattern identification, while classification techniques like linear discriminant analysis (LDA), k-nearest neighbor (*k*NN), or PLS-DA, represent supervised procedures. PLS proved to be more effective in a supervised workflow in terms of accuracy, while PCA is highly effective for revealing misalignments (22).

The fifth step represented model interpretation. The capability of a model is commonly determined by its accuracy, representing the ratio of accurately predicted specimens for a particular sample set. Since it is vulnerable to overfitting, the classification accuracy should only be employed as a reference. Furthermore, dividing the data set into training and validation information prevented overfitting. The classification success rate was impacted by sample collection (origin and number), PC selection, and model selection. For example, different success rates were acquired from LDA, *k*NN and PLS-DA with the same dataset (21).

### 2.2. Chemometric methods

Exploratory and classification methods are two kinds mostly used chemometric methods with GC-IMS data. Exploratory methods, such as PCA or HCA, are unsupervised and typically used for pattern recognition. Exploratory methods, or unsupervised statistical methods, are used to investigate data structures and visualize sample similarities. As can be seen in Table 1, almost all reviewed studies have employed PCA as the first step for data analysis. PLS-DA, LDA, and *k*NN are all supervised classification methods. For classification missions, the scores obtained by PCA are coupled with follow-up supervised methods to classify samples according to defined categories. Combined with PLS-DA, GC-IMS has classified agricultural products or foods according to their feature successfully, such as olive oil (23) and rice (24). Other supervised methods used in non-target screening with GC-IMS are orthogonal partial least-squares discriminant analysis (OPLS-DA), quadratic discriminant analysis (QDA), logistic regression, gradient boosting, decision tree classification, and soft independent modeling of class analogy (SIMCA) (25).

### 2.3. Insight into the combined GC-IMS and PLS-DA methodology

The reviewed literature on GC-IMS shows that PLS-DA is a commonly used, efficient chemometric tool for sample classification. Several factors were considered to increase the PLS-DA model accuracy.

The first factor involved the validation set. PCA was conducted before PLS-DA to capture natural sample variation in each class. The homogeneous sample set consisted of similar samples with low Hotelling’s *T*^2^ values. Previous research showed that optimal results were achieved via model training with samples distributed over the maximum area in the PCA score plot (26).

The second factor involved the training and validation set ratio. Like other multivariate approaches, PLS-DA was easily influenced by the training and validation set ratio. Previous studies tested the different ratios to determine the optimal value using different blind samples numbers (26). The results indicated that an accuracy of ≥85% was achieved when the number of training samples was least 1.8-fold higher than the blind samples. In summary, the number of training and blind samples should be equal.

The third factor involved the minimum number of training sets. Approaches have been conducted to find out minimum number of samples are necessary to train a PLS-DA model, which could predict the class of blind samples of different origin. A study involving Iberian ham revealed that a training set with about 450 samples was sufficient to develop a PLS-DA model, which could predict 300 blind samples (26). However, the number of training samples used in several other studies was considerably lower than 450.

The fourth factor involved the components of the training and validation sets. The accuracy of the PLS-DA model based on balanced training sets was higher than that involving biased training sets (26). Besides balance, the training set samples should include broad diversity regarding both classes. Therefore, the trained PLS-DA model needed to be reconstructed when including samples of a new origin.

The fifth factor involved marker or characteristic indicator selection. The crucial aroma substances were determined as markers according to the common criteria of a VIP score > 1 and *p* < 0.05.

The sixth factor involved the selection of whole spectral or pre-selected variables. Using the whole fingerprints during the data acquisition stage might be more time-efficient than employing pre-selected variables. However, using whole spectral fingerprints during the data processing stage was more time-consuming since large amounts of data demand massive computation power. Using pre-selected variables only requires GC-IMS spectral visual screening. Once the visual selection was completed, the parameters were saved and applied to all samples. In addition, a smaller amount of data requires less computing power for data processing. Moreover, the characteristic variables could be selected via VIP scores using the strength of the pre-selected features as data. Some studies involving dry-cured ham indicated that using markers enhanced the prediction results compared to fingerprints (26, 27).

## 3. Traceability and characteristic identification of GI products using GC-IMS

### 3.1. Agricultural products

#### 3.1.1. Rice

Hundreds of rice varieties are registered as GI products in China, the most prestigious of which is Wuchang rice (28), which is protected by a national standard (GB/T 19266-2008) by the Chinese government. The cultivar and production area are key rice price determinants (29), with that of GI Wuchang rice 20 times higher than non-GI products due to its high quality and unique flavor. The fingerprints of 53 rice samples from two main production areas (Wuchang and Guangxi) were extracted via GC-IMS, while their efficacy for the rapid identification of fragrant rice was verified (5). A three-dimensional GC-IMS map was employed to select the characteristic flavor substances from the rice specimens using an automatic threshold segmentation algorithm and image pretreatment, after which PCA and quadratic discriminant analysis (QDA) models were established to discriminate between fragrant rice from two areas. The PCA and QDA based on the GC-IMS data displayed a satisfactory identification rate and was applied to verify the authenticity of Wuchang rice.

#### 3.1.2. Red meat

Red meat (i.e., beef, pork, and lamb) is also a significant source of Chinese GI products. The quality of GI red meat is not only related to its origin but also to the animal breed, age, and feeding regimen (i.e., grass- or grain-fed). GC-IMS can also be used for GI red meat quality assessment.

The Jingyuan lamb is highly nutritious and approved as a GI product by the Chinese government. Since animal age plays a crucial role in lamb quality and is typically negatively correlated to eating quality (30), it is a decisive factor in market price. GI Jingyuan lambs usually include animals under 12 months to ensure a high eating quality. GC-IMS was applied to distinguish between and predict the ages of Jingyuan lambs at 2, 6, and 12 months (4). PCA was performed after GC-IMS data extraction, with the first principal component (PC1) contributing 67% to the cumulative variance, while the second principal component (PC2) accounted for 27%. The different ages of the lambs were clearly separated in the PCA plot. Therefore, combining GC-IMS with PCA could successfully classify the Jingyuan lambs at different months.

Indigenous Chinese pork, such as Beijing Heiliu and Laiwu black pork, is favored by consumers because of its unique, pleasant flavor related to complex reactions, such as lipid oxidation (31). Wu et al. (9) determined the flavor and fatty acid fingerprints in typical indigenous Chinese pork by combining GC-O-MS and GC-IMS with multivariate analysis. Here, 59 characteristic aroma compounds were selected according to a two-dimensional GC-IMS plot and used for PCA. PC1 contributed 41.6% to the variance contribution rate, while PC2 represented 25.9%, indicating that PC1 retains most of the fingerprint information of the samples. Furthermore, 79 VOCs were identified via GC-O-MS, of which 15 were selected as key odorants in Chinese indigenous pork. These results indicated that the pork aroma profiles were breed-dependent, which corresponded with a study by Zhang et al. (32).

#### 3.1.3. Fruit

Fruit is another major source of Chinese GI products. Grapes are consumed on a large scale worldwide due to their flavor and nutritional qualities. The sensory quality and consumer acceptability of grapes are significantly related to the place of origin (33). Molixiang grapes, also known as jasmine grapes, are widely consumed in China, and cultivated in most grape production areas (i.e., the Zhejiang, Liaoning, and Fujian provinces). One study combined GC-IMS with PCA for the regional determination of Molixiang grapes (10). Considerable variation was evident between the VOC fingerprints of grape samples from three regions extracted via GC-IMS, indicating that their aroma profiles largely depended on geographical location. PCA indicated that the geographical origins of the different samples were effectively differentiated. PC1 accounted for 53% of the variance, while PC2 represented 31%. Furthermore, the sensory assessment indicated that the grape aroma features were associated with the geographical origin (*p* ≤ 0.05). Moreover, the geographical marker compounds, including (*E*)-2-octenal, styrene, and benzaldehyde, were screened for quality assurance.

#### 3.1.4. Oil seed

Sesame is an oilseed extensively cultivated in Africa and Asia (34). China imports a significant amount of sesame seeds annually, primarily from Sudan, Ethiopia, Mozambique, and Togo, reaching 888.8 kilotons in 2020. Similar to other GI products, the sesame seed price is also related to their place of origin. Fingerprinting analysis (i.e., GC-IMS and ICP-MS) was coupled with chemometrics tools (PCA and PLS-DA) to differentiate between Chinese, Togolese, Sudanese, Mozambican, and Ethiopian sesame seeds (11). The sesame seed samples yielded a total of 44 VOCs, while the aroma profiles varied substantially according to the different areas of origin. The GC-IMS volatile data were used for PCA, with the first two principal components accounting for 71.95% of the total variance. The volatile data were also processed using the PLS-DA model, with a 0.92 *R*^2^ value showing excellent fitting capacity and a Q^2^ value of 0.72, indicating a reliable prediction capacity towards the new dataset. In addition, the variable importance in projection (VIP) yielded 12 VOCs as markers to classify and differentiate the Chinese, Togolese, Sudanese, Mozambican, and Ethiopian sesame seeds. The results indicated that combining GC-IMS with PLS-DA shows promise for identifying geographical sesame seed origins.

#### 3.1.5. Honey

Botanical and geographical honey origins are considered crucial for the sustainable, ethical development of the bee industry. Winter honey is harvested by *Apis cerena* during the winter from wild *Eurya* spp. of the Theaceae family and *Schefflera actinophylla* (Endl.) Harms (35). High-quality winter honey is favored by Chinese consumers due to its unique flavor. During summer, *Sapium* honey is derived from *Sapium sebiferum* (L.) Roxb of the Asclepiadaceae family. Unlike winter honey, consumer acceptance of *Sapium* honey is lower due to its slightly coarse crystallization, sour taste, and low concentration. Wang et al. (12) established a reliable, rapid model to differentiate between *Sapium*, winter, and contaminated honey using the GC-IMS data. Consequently, combining GC-IMS with PCA and PLS-DA clearly distinguished between winter and *Sapium* honey. During PCA, PC1 accounted for 57.9% of the total variability, while PC2 represented 14.5%. In addition, the honey samples mentioned above were clustered into different groups using PCA. PLS-DA yielded an *R*^2^X value of 0.72, an *R*^2^Y value of 0.88, and a Q^2^ value of 0.84, highlighting the excellent model fitting capability and predictability. The winter and *Sapium* honey markers were screened and confirmed by combining the GC-IMS database with PLS-DA.

#### 3.1.6. Fish

Salmonid flavor is impacted by the species, place of origin, and living conditions (36). Despite similar quality and nutritional properties, the Atlantic salmon price is double that of Rainbow trout in the Chinese market (13). Due to the complexity of salmonid fish fillet sources, Atlantic salmon label fraud is difficult for consumers to identify and the government to regulate. GC-IMS and intelligent sensory technology (E-nose, electronic tongue) were combined to screen flavor markers in the two salmonid species mentioned above from China and Chile (13). Flavor fingerprints were extracted via GC-IMS and then subjected to PCA. PC1 accounted for 58% of the cumulative variance, while PC2 contributed 19%. Samples belonged to different class scattered, respectively, in PCA plot, demonstrating that PCA could be used to classify the salmonid origins and species according to their aroma profiles. HCA was employed to identify the two main clusters in the heat map. Furthermore, the GC-IMS and E-nose results were consistent. Therefore, combining GC-IMS with PCA can distinguish between the different places of origin of salmonids to protect GI products during the international trading process.

#### 3.1.7. Spice

*Zanthoxylum armatum* DC and *Zanthoxylum bungeanum* Maxim., also known as huajiao or Sichuan pepper, is highly regarded in China due to its unique taste and distinctive aroma (37). Huajiao, with a unique perception known as “ma” in Chinese, is typically used in Sichuan cuisine as a ground powder or whole (38). Huajiao is cultivated in various Chinese regions with diverse climates, leading to distinct differences between huajiao crops. Eight red and green huajiao species verified as GI Chinese products were analyzed using an E-nose, GC-IMS, and SPME-GC-MS (14). Sixty-two peaks denoting characteristic aromas were determined via GC-IMS. The ability of the GC-IMS and traditional GC-MS, whose data sets both coupled with PCA and PLS-DA, were compared on classification of huajiao from different origins in this research. Here, 61.45% of the cumulative variance was represented by PC1 and PC2 in the PCA bi-plot of the GC-MS data set, while the GC-IMS value was slightly lower at 66.91%. The PLS-DA model constructed using the GC-IMS and GC-MS data effectively classified the different huajiao places of origin. However, according to the VIP scores, these two methods produced four and eight VOC biomarkers, respectively. These results indicated that combining GC-IMS and PLS-DA could be useful for GI huajiao traceability.

### 3.2. Traditional Chinese food products

#### 3.2.1. Tea

Tea is a valuable GI product, the quality of which is related to its place of origin, harvesting season, and aroma type (39). The sale of fake GI tea products to increase profits severely impacts brand protection and violates consumer rights (40).

Fu brick tea is a well-known Chinese GI product popular with consumers worldwide because of its unique aroma and health advantages, and it is cultivated in various Chinese regions, including Guizhou, Hunan, Guangxi, Zhejiang, and Shaanxi Provinces. GC-IMS and GC-MS were employed to determine the aroma profiles of five Fu brick tea samples from these areas (6), producing 93 and 63 VOCs, respectively. The GC-IMS fingerprints were used to construct PCA and HCA models. The PCA map indicated a clear separation between the places of origin of the five samples. Furthermore, the crucial aroma compounds identified via PLS-DA were used to distinguish between the geographical areas of the five Fu brick tea samples. The VIP scores indicated 29 marker VOCs, while the odor activity value (OAV) showed that 27 were critical for overall flavor profiles of the samples. Fifteen of these VOCs were effectively applied to differentiate between the aroma profiles of the different Fu brick tea samples.

Aroma type represents a key factor during tea quality evaluation (41). The black tea aromas were divided into floral, sweet, faint scent, and fruity, while the aroma compounds were systematically analyzed via GC-IMS, an E-Nose, and the OAV (15). GC-IMS identified 38 aroma compounds, 15 of which were key compounds with OAVs exceeding 1 in three black tea samples, including ethyl 2-methylpentanoate, 3-methylbutanal, (*E*)-2-nonenal, and linalool. PLS-DS effectively distinguished between these aroma types, using the GC-IMS and E-nose datasets and robust model parameters, showing consistent results. Furthermore, 18 aroma compounds with VIP values >1.0 were selected as potential biomarkers.

#### 3.2.2. Traditional Chinese meat products

Since traditional Chinese dry-cured ham has a unique flavor, its sensory characteristics vary significantly between regions due to variations during the manufacturing process, which involves salting, pressing, drying, and ripening (42). Well-known Chinese dry-cured ham includes Jinhua (from Zhejiang), Rugao (from Jiangsu), Xuanwei, Nuodeng, Sanchuan, Saba (from Yunan), Mianning (from Sichuan), Xuanen (from Hubei), and Wanhua (from Anhui), most of which have GI status.

The VOCs of Chinese dry-cured ham from the Xuanen, Wanhua, Sanchuan, Saba, Nuodeng, and Mianning regions were analyzed via two-dimensional gas chromatography-mass spectrometry-time-of-flight-mass spectrometry (GC × GC-ToF-MS) and GC-IMS (17). GC × GC-ToF-MS identified 265 VOCs, which was over five times more than the 45 detected via GC-IMS. Multiple factor analysis (MFA) and PCA were employed for sample flavor profile visualization and differentiation, producing similar results regardless of whether GC × GC-ToF-MS or GC-IMS data were used. Therefore, GC-IMS was a reliable method for classifying dry-cured ham from different regions.

Jinhua ham, produced from the famous local Liangtouwu pig breed, was approved as a GI product in 2001 by the Chinese government. The unique flavor of Jinhua ham is popular among Chinese consumers and is related to the production process during which the pork is salted, washed, dried, shaped, ripened, and post-ripened (42). GC-IMS was utilized for the reliable, rapid recognition of Jinhua ham samples during different stages of ripening (18), identifying 37 VOCs, which included dimers and monomers. The PCA plot indicated clear separation between the ham samples at various aging times. PC1 represented 37.38% of the sample variance, while PC2 denoted 22.32%. These results indicated that combining GC-IMS with multivariance analysis (i.e., PCA) facilitated the rapid identification of Jinhua ham flavor profiles, providing information related to aging time.

### 3.3. Traditional Chinese liquor

#### 3.3.1. Chinese yellow wine

Traditional Chinese yellow wine, derived from glutinous rice and wheat, was originally developed in Shaoxing, China. It has been popular with Chinese customers for centuries (43), providing significant commercial value as a GI product. The fraudulent use of the Shaoxing brand for producing many yellow wines in non-Shaoxing areas necessitates the development of a rapid identification technique to verify authenticity (44). GC-IMS differentiated between 122 Chinese yellow wines from Shandong, Hubei, and Shaoxing (7). The characteristic peaks were visualized using a simple color-mixing method. The VOCs were determined via a library search, while the peak height values were used as data sets for further chemometric analysis. PCA revealed significant differences between the samples, while QDA was used for wine sample classification, displaying a 95.35% accuracy rate for the prediction set. Consequently, combining the flavor data set of GC-IMS with PCA and QDA could effectively determine Chinese yellow wine authenticity.

#### 3.3.2. Chinese Baijiu

Baijiu, famous for its unique flavor, is a distilled spirit in production for more than 2,000 years (45). Most prominent Baijiu brands have GI status with substantial annual output value, resulting in significant fraud regarding factors such as the aging duration of the product. The price of Baijiu is highly associated with the duration of aging, an extremely time-consuming stage, and essential for high-quality Baijiu production. Therefore, developing an effective method for determining Baijiu aging time is necessary to protect consumers against fake products (46). GC-IMS was employed to analyze 39 Baijiu specimens from various production years (1998–2019), obtained from pottery jars in workshops (19). Partial least squares regression (PLSR) analysis was performed utilizing the signal peaks (212) and identified compounds (93) as data sets to establish two valid models. The accuracy of the models in determining the aging time of the Baijiu samples depended on the root mean square error of prediction (RMSEP) and the fit value (*R*^2^). Nineteen of the 93 identified compounds displayed VIP scores >1 and were selected as markers.

## 4. Data fusion

Data fusion, denoting multiple data source integration, may enhance model reliability and accuracy and reduce interference and error rates, and can be characterized as low- (data-level), mid- (feature-level), and high-level (decision-level) (47). The GC-IMs included a data fusion strategy to assess olive oil quality. A recent study used liquid chromatography-high resolution mass spectrometry (LC-MS), GC-IMS, and an E-nose to identify extra virgin olive oil (EVOO) and soft, refined oil (SROO) mixtures (23). Here, 43 EVOO samples were collected from a market, while 18 adulterated oils were created by mixing SROO and EVOO. Data fusion was performed at low- and mid-levels, while PLS-DA occurred using the merged data sets, and a support vector machine (SVM) model was developed utilizing the potential characteristic variables. Combining PLS-DA with SVM using the merged datasets demonstrated that data fusion at a low-level significantly improved the classification precision compared to the individual techniques.

## 5. Summary and prospects

Combining GC-IMS with a chemometric method can efficiently and rapidly determine the traceability and characteristics of GI products due to low maintenance and time efficiency. This method can be used for classification and authentication, such as determining places of origin, harvest seasons, animal age, and feeding regimens, and can be applied to most GI and food products in China like rice, red meat, fruit, oil seed, honey, fish, spice, tea, dry-cured ham, Chinese yellow wine, and Baijiu. Furthermore, the general workflow of these methods is summarized, including sample collection, data acquisition and processing, and model construction and interpretation. Considering that PLS-DA is a commonly employed chemometric technique, several factors related to model construction have been concluded to increase the accuracy. Furthermore, data fusion is also reviewed, proving an effective way to increase accuracy.

However, there are still challenges in GC-IMS approach. Due to the inadequate database, some VOC could not be identified by GC-IMS. To improve the application of GC-IMS technology on classification and authentication of GI products, following aspects need to be researched in the future. First, a comprehensive and extensive database of VOC for GC-IMS need to be established, which should be as efficient as database of GC-MS. Second, databases of fingerprint of aroma profile for each specific GI agricultural product or food need to be developed. Without this fingerprint database, the widespread use of GC-IMS approach is not possible. Third, data fusion strategy needs to be studied intensively, as GC-IMS only provides aroma characteristics. Combined with other techniques, panoramic view of nutrition and sensory features of GI agricultural products and food can be presented.

## Author contributions

HZ: original draft, writing, and review and editing. DZ: validation and investigation. JS: supervision and project administration. All authors contributed to the article and approved the submitted version.

## Conflict of interest

The authors declare that the research was conducted in the absence of any commercial or financial relationships that could be construed as a potential conflict of interest.

## Publisher’s note

All claims expressed in this article are solely those of the authors and do not necessarily represent those of their affiliated organizations, or those of the publisher, the editors and the reviewers. Any product that may be evaluated in this article, or claim that may be made by its manufacturer, is not guaranteed or endorsed by the publisher.

## References

[1] Wang S Chen H Sun B. Recent progress in food flavor analysis using gas chromatography-ion mobility spectrometry (GC-IMS). Food Chem. (2020) 315:126158. doi: 10.1016/j.foodchem.2019.126158 32014672

[2] Borsdorf H Eiceman GA. Ion mobility spectrometry: principles and applications. Appl Spectrosc Rev. (2006) 41:323–75. doi: 10.1080/05704920600663469

[3] Furuhashi T Okuda K. Application of GC/MS soft ionization for isomeric biological compound analysis. Crit Rev Anal Chem. (2017) 47:438–53. doi: 10.1080/10408347.2017.1320215 28441028

[4] Wang F Gao Y Wang H Xi B He X Yang X. Analysis of volatile compounds and flavor fingerprint in Jingyuan lamb of different ages using gas chromatography-ion mobility spectrometry (GC-IMS). Meat Sci. (2021) 175:108449. doi: 10.1016/j.meatsci.2021.108449 33550158

[5] Chen T Li H Chen X Wang Y Cheng Q Qi X. Construction and application of exclusive flavour fingerprints from fragrant rice based on gas chromatography – ion mobility spectrometry (GC-IMS). Flavour Fragr J. (2022) 37:345–53. doi: 10.1002/ffj.3716

[6] Xiao Y Huang Y Chen Y Xiao L Zhang X Yang C. Discrimination and characterization of the volatile profiles of five Fu brick teas from different manufacturing regions by using HS-SPME/GC-MS and HS-GC-IMS. Curr. Res. Food Sci. (2022) 5:1788–807. doi: 10.1016/j.crfs.2022.09.024 36268133 PMC9576573

[7] Chen T Qi X Chen M Lu D Chen B. Discrimination of Chinese yellow wine from different origins based on flavor fingerprint. Acta Chromatogr. (2020) 32:139–44. doi: 10.1556/1326.2019.00613

[8] Rodriguez-Hernandez P Martin-Gomez A Cardador MJ Amaro MA Arce L Rodriguez-Estevez V. Geographical origin, curing plant and commercial category discrimination of cured Iberian hams through volatilome analysis at industry level. Meat Sci. (2023) 195:108989–9. doi: 10.1016/j.meatsci.2022.108989 36228509

[9] Wu W Zhan J Tang X Li T Duan S. Characterization and identification of pork flavor compounds and their precursors in Chinese indigenous pig breeds by volatile profiling and multivariate analysis. Food Chem. (2022) 385:132543. doi: 10.1016/j.foodchem.2022.132543 35287104

[10] Feng T Sun J Song S Wang H Yao L Sun M. Geographical differentiation of Molixiang table grapes grown in China based on volatile compounds analysis by HS-GC-IMS coupled with PCA and sensory evaluation of the grapes. Food Chem X. (2022a) 15:100423. doi: 10.1016/j.fochx.2022.100423 36211739 PMC9532774

[11] Mi S Wang Y Zhang X Sang Y Wang X. Authentication of the geographical origin of sesame seeds based on proximate composition, multi-element and volatile fingerprinting combined with chemometrics. Food Chem. (2022) 397:133779. doi: 10.1016/j.foodchem.2022.133779 35914458

[12] Wang X Yang S He J Chen L Zhang J Jin Y. A green triple-locked strategy based on volatile-compound imaging, chemometrics, and markers to discriminate winter honey and sapium honey using headspace gas chromatography-ion mobility spectrometry. Food Res Int. (2019) 119:960–7. doi: 10.1016/j.foodres.2019.01.004 30884736

[13] Duan Z Dong S Dong Y Gao Q. Geographical origin identification of two salmonid species via flavor compound analysis using headspace-gas chromatography-ion mobility spectrometry combined with electronic nose and tongue. Food Res Int. (2021) 145:110385. doi: 10.1016/j.foodres.2021.110385 34112388

[14] Feng X Wang H Wang Z Huang P Kan J. Discrimination and characterization of the volatile organic compounds in eight kinds of huajiao with geographical indication of China using electronic nose, HS-GC-IMS and HS-SPME-GC-MS. Food Chem. (2022b) 375:131671. doi: 10.1016/j.foodchem.2021.131671 34865919

[15] Yang Y Zhu H Chen J Xie J Shen S Deng Y. Characterization of the key aroma compounds in black teas with different aroma types by using gas chromatography electronic nose, gas chromatography-ion mobility spectrometry, and odor activity value analysis. LWT. (2022) 163:113492. doi: 10.1016/j.lwt.2022.113492

[16] Liu H Xu Y Wu J Wen J Yu Y An K. GC-IMS and olfactometry analysis on the tea aroma of Yingde black teas harvested in different seasons. Food Res Int. (2021) 150:110784. doi: 10.1016/j.foodres.2021.110784 34865799

[17] Li W Chen YP Blank I Li F Li C Liu Y. GC × GC-ToF-MS and GC-IMS based volatile profile characterization of the Chinese dry-cured hams from different regions. Food Res Int. (2021b) 142:110222. doi: 10.1016/j.foodres.2021.110222 33773696

[18] Liu D Bai L Feng X Chen YP Zhang D Yao W. Characterization of Jinhua ham aroma profiles in specific to aging time by gas chromatography-ion mobility spectrometry (GC-IMS). Meat Sci. (2020) 168:108178. doi: 10.1016/j.meatsci.2020.108178 32417671

[19] Chen S Lu J Qian M He H Li A Zhang J. Untargeted headspace-gas chromatography-ion mobility spectrometry in combination with chemometrics for detecting the age of Chinese liquor (Baijiu). Foods. (2021) 10:2888. doi: 10.3390/foods10112888 34829169 PMC8621296

[20] Hernández-Mesa M Ropartz D García-Campaña AM Rogniaux H Dervilly-Pinel G Le Bizec B. Ion mobility spectrometry in food analysis: principles, current applications and future trends. Molecules. (2019) 24:2706. doi: 10.3390/molecules24152706 31349571 PMC6696101

[21] Gerhardt N Birkenmeier M Schwolow S Rohn S Weller P. Volatile-compound fingerprinting by headspace-gas-chromatography ion-mobility spectrometry (HS-GC-IMS) as a Benchtop alternative to ^1^H NMR profiling for assessment of the authenticity of honey. Anal Chem. (2018) 90:1777–85. doi: 10.1021/acs.analchem.7b03748 29298045

[22] Christmann J Rohn S Weller P. Finding features – variable extraction strategies for dimensionality reduction and marker compounds identification in GC-IMS data. Food Res Int. (2022) 161:111779. doi: 10.1016/j.foodres.2022.111779 36192933

[23] Tata A Massaro A Damiani T Piro R Dall'Asta C Suman M. Detection of soft-refined oils in extra virgin olive oil using data fusion approaches for LC-MS, GC-IMS and FGC-Enose techniques: the winning synergy of GC-IMS and FGC-Enose. Food Control. (2022) 133:108645. doi: 10.1016/j.foodcont.2021.108645

[24] Zhang X Dai Z Fan X Liu M Ma J Shang W. A study on volatile metabolites screening by HS-SPME-GC-MS and HS-GC-IMS for discrimination and characterization of white and yellowed rice. Cereal Chem. (2020) 97:496–504. doi: 10.1002/cche.10264

[25] Capitain C Weller P. Non-targeted screening approaches for profiling of volatile organic compounds based on gas chromatography-ion mobility spectroscopy (GC-IMS) and machine learning. Molecules. (2021) 26:5457. doi: 10.3390/molecules26185457 34576928 PMC8468721

[26] Martín-Gómez A Rodríguez-Hernández P Cardador MJ Vega-Márquez B Rodríguez-Estévez V Arce L. Guidelines to build PLS-DA chemometric classification models using a GC-IMS method: dry-cured ham as a case of study. Talanta Open. (2023) 7:100175. doi: 10.1016/j.talo.2022.100175

[27] Arroyo-Manzanares N Martín-Gómez A Jurado-Campos N Garrido-Delgado R Arce C Arce L. Target vs spectral fingerprint data analysis of Iberian ham samples for avoiding labelling fraud using headspace – gas chromatography-ion mobility spectrometry. Food Chem. (2018) 246:65–73. doi: 10.1016/j.foodchem.2017.11.008 29291880

[28] Song HX Lu BY Ye CH Li J Zhu ZW Zheng LF. Fraud vulnerability quantitative assessment of Wuchang rice industrial chain in China based on AHP-EWM and ANN methods. Food Res Int. (2021) 140:109805. doi: 10.1016/j.foodres.2020.109805 33648162

[29] Suzuki Y. Achieving food authenticity and traceability using an analytical method focusing on stable isotope analysis. Anal Sci. (2021) 37:189–99. doi: 10.2116/analsci.20SAR14 33229826

[30] Payne CE Pannier L Anderson F Pethick DW Gardner GE. Lamb age has little impact on eating quality. Foods. (2020) 9:187. doi: 10.3390/foods9020187 32069988 PMC7073923

[31] Li J Zhang J Yang Y Zhu J He W Zhao Q. Comparative characterization of lipids and volatile compounds of Beijing Heiliu and Laiwu Chinese black pork as markers. Food Res Int. (2021a) 146:110433. doi: 10.1016/j.foodres.2021.110433 34119242

[32] Zhang Y Zhang YJ Li H Guo TR Jia JL Zhang PC. Comparison of nutrition and flavor characteristics of five breeds of pork in China. Foods. (2022) 11:2704. doi: 10.3390/foods11172704 36076889 PMC9455266

[33] Ferrero-del-Teso S Suárez A Ferreira C Perenzoni D Arapitsas P Mattivi F. Modeling grape taste and mouthfeel from chemical composition. Food Chem. (2022) 371:131168. doi: 10.1016/j.foodchem.2021.131168 34601211

[34] Kim SY Kim E Shin BK Seo JA Kim YS Lee D. NMR-based metabolic profiling discriminates the geographical origin of raw sesame seeds. Food Control. (2020) 112:107113. doi: 10.1016/j.foodcont.2020.107113

[35] Huang S Ni H Wu L Yang Y. Differentiating the optimal pH of amylase for the analysis of commercial mesophilic amylase in hundred nectars and Scheffleraoctophylla (Lour.) harms honey. J. Chin. Inst. Food Sci. Technol. (2014) 14:266–72. doi: 10.16429/j.1009-7848.2014

[36] Mabuchi R Ishimaru A Tanaka M Kawaguchi O Tanimoto S. Metabolic profiling of fish meat by GC-MS analysis, and correlations with taste attributes obtained using an electronic tongue. Meta. (2019) 9:1. doi: 10.3390/metabo9010001 PMC6358880 30577613

[37] Ivane NMA Haruna SA Zekrumah M Elyse FKR Hassan MO Hashim SBH. Composition, mechanisms of tingling paresthesia, and health benefits of Sichuan pepper: a review of recent progress. Trends Food Sci Technol. (2022) 126:1–12. doi: 10.1016/j.tifs.2022.05.012

[38] Deng S Rong H Tu H Zheng B Mu X Zhu L. Molecular basis of neurophysiological and antioxidant roles of Szechuan pepper. Biomed Pharmacother. (2019) 112:108696. doi: 10.1016/j.biopha.2019.108696 30818139

[39] Cai HL Zhong ZH Li ZM Zhang XJ Fu HW Yang BX. Metabolomics in quality formation and characterisation of tea products: a review. Int J Food Sci Technol. (2022) 57:4001–14. doi: 10.1111/ijfs.15767

[40] Shuai M Peng C Niu H Shao D Hou R Cai H. Recent techniques for the authentication of the geographical origin of tea leaves from camellia sinensis: a review. Food Chem. (2022) 374:131713. doi: 10.1016/j.foodchem.2021.131713 34920400

[41] Zhai XT Zhang L Granvogl M Ho CT Wan XC. Flavor of tea (*Camellia sinensis*): a review on odorants and analytical techniques. Compr Rev Food Sci Food Saf. (2022) 21:3867–909. doi: 10.1111/1541-4337.12999 35810334

[42] Li FY Feng X Zhang DN Li CB Xu XL Zhou GH. Physical properties, compositions and volatile profiles of Chinese dry-cured hams from different regions. J. Food Meas. Charact. (2020) 14:492–504. doi: 10.1007/s11694-019-00158-9

[43] Peng Q Zheng HJ Meng K Zhu YM Zhu WX Zhu HY. The way of Qu-making significantly affected the volatile flavor compounds in Huangjiu (Chinese rice wine) during different brewing stages. Food Sci Nutr. (2022b) 10:2255–70. doi: 10.1002/fsn3.2835 35844911 PMC9281927

[44] Peng Q Chen JL Meng K Zheng HJ Chen GQ Xu X. Rapid detection of adulteration of glutinous rice as raw material of Shaoxing Huangjiu (Chinese Rice wine) by near infrared spectroscopy combined with chemometrics. J Food Compos Anal. (2022a) 111:104563. doi: 10.1016/j.jfca.2022.104563

[45] Xu YQ Zhao JR Liu X Zhang CS Zhao ZG Li XT. Flavor mystery of Chinese traditional fermented Baijiu: the great contribution of ester compounds. Food Chem. (2022) 369:130920. doi: 10.1016/j.foodchem.2021.130920 34461518

[46] Liu QR Zhang XJ Zheng L Meng LJ Liu GQ Yang T. Machine learning based age-authentication assisted by chemo-kinetics: case study of strong-flavor Chinese Baijiu. Food Res Int. (2023) 167:112594. doi: 10.1016/j.foodres.2023.112594 37087223

[47] Borràs E Ferré J Boqué R Mestres M Aceña L Busto O. Data fusion methodologies for food and beverage authentication and quality assessment – a review. Anal Chim Acta. (2015) 891:1–14. doi: 10.1016/j.aca.2015.04.042 26388360

